# Supplementary material for: Validation and Psychometric Properties of the German Operational and Organizational Police Stress Questionnaires
Source: Int J Environ Res Public Health. 2023 Sep 26;20(19):6831. doi: 10.3390/ijerph20196831 (PMC10572357; doi:10.3390/ijerph20196831)
Supplement: Supplementary file 1 [file ijerph-20-06831-s001.zip › ijerph-2588696-supplementary.pdf]

Validation and Psychometric Properties of the German Operational and Organizational Police Stress Questionnaires

Lauriane Willemin-Petignat, Royce Anders, Sabrina Ogi, Benjamin Putois

Table S1 Exploratory Factory Analysis of the PSQ-G

Items		Operational	Organizational	Communalities
PSQ-G-Op-1	*Shift work*<br>Schichtarbeit	0.63		0.42
PSQ-G-Op-2	*Working alone at night*<br>Nachts alleine arbeiten	0.46		0.25
PSQ-G-Op-3	*Over-time demand*<br>Überstundenforderung	0.57	0.34	0.43
PSQ-G-Op-4	*Risk of being injured on the job*<br>Risiko, bei der Arbeit verletzt zu werden	0.52		0.34
PSQ-G-Op-5	*Work related activities on days off (e.g., court, community events)*<br>Arbeitsbezogene Aktivitäten an freien Tagen (z. B. Gericht, Gemeindeveranstaltungen)	0.45		0.29
PSQ-G-Op-6	*Traumatic events (e.g., MVA, domestics, death, injury)*<br>Traumatische Ereignisse (z. B. Motorfahrzeugunfall, Häusliche Gewalt, Tod, Verletzungen)	0.45		0.27
PSQ-G-Op-7	*Managing your social life outside of work*<br>Management des soziales Lebens ausserhalb der Arbeit	0.65		0.46
PSQ-G-Op-8	*Not enough time available to spend with friends and family*<br>Zu wenig Zeit für Freunde und Familie	0.71		0.57
PSQ-G-Op-9	*Paperwork*<br>Papierkram		**0.66**	0.49
PSQ-G-Op-10	*Eating healthy at work*<br>Gesunde Ernährung bei der Arbeit	0.54		0.37
PSQ-G-Op-11	*Finding time to stay in good physical condition*<br>Die Zeit finden, um in guter körperlicher Verfassung zu bleiben	0.6		0.42
PSQ-G-Op-12	*Fatigue (e.g. shift work, over-time)*<br>Ermüdung (z. B. Schichtarbeit, Überstunden)	0.69	0.3	0.57

PSQ-G-Op-13	*Occupation-related health issues (e.g. back pain)*<br>Berufsbedingte Gesundheitsprobleme (z. B. Rückenschmerzen)	0.52	0.31	0.37
PSQ-G-Op-14	*Lack of understanding from family and friends about your work*<br>Mangelndes Verständnis von Familie und Freunde für die Polizeiarbeit	0.54		0.32
PSQ-G-Op-15	*Making friends outside the job*<br>Freunde ausserhalb der Arbeit finden	0.6		0.38
PSQ-G-Op-16	*Upholding a "higher image" in public*<br>Aufrechterhaltung eines «besseren Images» in der Öffentlichkeit	0.54	0.34	0.41
PSQ-G-Op-17	*Negative comments from the public*<br>Negative Kommentare aus der Öffentlichkeit	0.48	0.33	0.34
PSQ-G-Op-18	*Limitations to your social life (e.g. who your friends are, where you socialize)*<br>Einschränkung im sozialen Leben (z. B. Wer Ihre Freunde sind, wo Sie sich treffen)	0.71		0.53
PSQ-G-Op-19	*Feeling like you are always on the job*<br>Das Gefühl, ständig im Einsatz zu sein	0.66	0.34	0.54
PSQ-G-Op-20	*Friends / family feel the effects of the stigma associated with your job*<br>Freunde/Familie spüren die Auswirkung des Berufsstigmas	0.66	0.3	0.53
PSQ-G-Org-1	*Dealing with co-workers*<br>Umgang mit Arbeitskollegen		0.34	0.15
PSQ-G-Org-2	*The feeling that different rules apply to different people (e.g., favouritism)*<br>Das Gefühl, dass für verschiedene Menschen unterschiedliche Regeln gelten (z. B. Favoritismus/Bevorzugung)		0.42	0.26
PSQ-G-Org-3	*Feeling like you always have to prove yourself to the organization*<br>Das Gefühl, sich dem Arbeitsgeber ständig beweisen zu müssen	0.4	0.49	0.4
PSQ-G-Org-4	*Excessive administrative duties*<br>Übermässige administrative Aufgaben		0.72	0.53
PSQ-G-Org-5	*Constant changes in policy/legislation*<br>Ständige Änderungen in der Politik/Gesetzgebung		0.58	0.38
PSQ-G-Org-6	*Staff shortages*<br>Personalmangel		0.58	0.42

PSQ-G-Org-7	*Bureaucratic red tape*<br>Bürokratischer Aufwand		0.77	0.61
PSQ-G-Org-8	*Too much computer work*<br>Zu viel Computerarbeit		0.62	0.41
PSQ-G-Org-9	*Lack of training on new equipment*<br>Mangelndes Training mit neuer Ausrüstung		0.41	0.22
PSQ-G-Org-10	*Perceived pressure to volunteer free time*<br>Wahrgenommener Druck, sich in der Freizeit engagieren zu müssen	0.44	0.31	0.29
PSQ-G-Org-11	*Dealing with supervisors*<br>Umgang mit Vorgesetzten		0.48	0.3
PSQ-G-Org-12	*Inconsistent leadership style*<br>Inkonsistenter Führungsstil		0.49	0.31
PSQ-G-Org-13	*Lack of resources*<br>Ressourcenmangel	0.31	0.62	0.48
PSQ-G-Org-14	*Unequal sharing of work responsibilities*<br>Ungleiche Aufteilung der Arbeitsaufgaben		0.61	0.46
PSQ-G-Org-15	*If you are sick or injured your co-workers seem to look down on you*<br>Wenn Sie krank oder verletzt sind, scheinen Mitarbeitende auf Sie herabzuschauen	**0.41**		0.25
PSQ-G-Org-16	*Leaders over-emphasise the negatives (e.g. supervisor evaluations, public complaints)*<br>Führungskräfte betonen zu sehr das Negative (z. B. Bewertungen von Vorgesetzten, öffentliche Beschwerden)	0.34	0.46	0.33
PSQ-G-Org-17	*Internal investigations*<br>Interne Ermittlungen		0.35	0.2
PSQ-G-Org-18	*Dealing the court system*<br>Umgang mit dem Gerichtssystem		0.45	0.25
PSQ-G-Org-19	*The need to be accountable for doing your job*<br>Das Bedürfnis, für seine Arbeit verantwortlich zu sein	0.37	0.43	0.32
PSQ-G-Org-20	*Inadequate equipment*<br>Unzureichende Ausrüstung		0.38	0.22
SS Loadings		8.17	6.92	
Explained Variance		20.4%	17.3%	

Note: N = 2314; Bolded values highlighted items who cross in the wrong factor.
